# Unraveling the Functional Significance of Unstructured Regions in G Protein-Coupled Receptors

**DOI:** 10.3390/biom13101431

**Published:** 2023-09-22

**Authors:** Roberto Maggio, Irene Fasciani, Francesco Petragnano, Maria Francesca Coppolino, Marco Scarselli, Mario Rossi

**Affiliations:** 1 Department of Biotechnological and Applied Clinical Sciences, University of L’Aquila, 67100 L’Aquila, Italy; irene.fasciani@univaq.it (I.F.); francesco.petragnano@univaq.it (F.P.); mario.rossi@univaq.it (M.R.); 2 Department of Life, Health and Environmental Sciences, University of L’Aquila, 67100 L’Aquila, Italy; mariafrancesca.coppolino@univaq.it; 3 Department of Translational Research and New Technologies in Medicine and Surgery, University of Pisa, 56126 Pisa, Italy; marco.scarselli@unipi.it

**Keywords:** G protein-coupled receptor, unstructured protein, third cytoplasmic loop, C-terminus, domain swapping

## Abstract

Unstructured regions in functional proteins have gained attention in recent years due to advancements in informatics tools and biophysical methods. G protein-coupled receptors (GPCRs), a large family of cell surface receptors, contain unstructured regions in the form of the i3 loop and C-terminus. This review provides an overview of the functional significance of these regions in GPCRs. GPCRs transmit signals from the extracellular environment to the cell interior, regulating various physiological processes. The i3 loop, located between the fifth and sixth transmembrane helices, and the C-terminus, connected to the seventh transmembrane helix, are determinant of interactions with G proteins and with other intracellular partners such as arrestins. Recent studies demonstrate that the i3 loop and C-terminus play critical roles in allosterically regulating GPCR activation. They can act as autoregulators, adopting conformations that, by restricting G protein access, modulate receptor coupling specificity. The length and unstructured nature of the i3 loop and C-terminus provide unique advantages in GPCR interactions with intracellular protein partners. They act as “fishing lines”, expanding the radius of interaction and enabling GPCRs to tether scaffolding proteins, thus facilitating receptor stability during cell membrane movements. Additionally, the i3 loop may be involved in domain swapping between GPCRs, generating novel receptor dimers with distinct binding and coupling characteristics. Overall, the i3 loop and C-terminus are now widely recognized as crucial elements in GPCR function and regulation. Understanding their functional roles enhances our comprehension of GPCR structure and signaling complexity and holds promise for advancements in receptor pharmacology and drug development.

## 1. Introduction

The presence of large unstructured regions, without a well-defined globular structure, is surprisingly common in functional proteins [1,2]. While the existence of functional unstructured proteins, such as polypeptide hormones, has been acknowledged for many years [3], the recognition of the functional significance of intrinsically disordered regions of proteins in processes like transcriptional regulation, translation, and cellular signal transduction is relatively recent. This newfound recognition is the result of employing new informatic tools, such as large-scale sequencing and gene-based functional analysis that provide extensive sequence data to identify intrinsically unstructured sequences within proteins [4] and computational algorithms used to predict and analyze intrinsically disordered regions based on sequence features, such as amino acid composition, flexibility, and predicted disorder propensity. Furthermore, while traditional crystal-structure analysis is limited in capturing structured states, nuclear magnetic resonance (NMR) spectroscopy [5] together with other biophysical methods such as small-angle X-ray scattering (SAXS) [6], single-molecule fluorescence [7], electron paramagnetic resonance (EPR) [8], and mass spectrometry [9] have advanced in sensitivity and resolution, enabling the study of the structural propensities and dynamics of disordered proteins in solution. NMR spectroscopy, in particular, provides valuable information on the conformational behavior and flexibility of intrinsically disordered regions [5]. This review article focuses on the unstructured region of G protein-coupled receptors (GPCRs) and provides an overview of their general characteristics. It summarizes the functional roles that these regions can play in GPCRs, and explores the advantages and biological consequences of the presence of regions that fold upon binding of their endogenous ligands or function as flexible linkers within cellular machinery allowing the receptor to maintain contact with other proteins.

## 2. The GPCR Family

GPCRs are a large family of cell surface receptors involved in a wide range of physiological processes. They are integral membrane proteins that classically transmit signals from the extracellular environment to the inside of the cell, although recently they have been demonstrated to play a significant role within intracellular compartments as well [10]. GPCRs are composed of seven transmembrane helices connected by intracellular and extracellular loops, with an extracellular amino (N)-terminus and an intracellular carboxyl (C)-terminal end. When a ligand (such as a hormone or neurotransmitter) binds to the receptor, it activates a G protein or binds a scaffolding protein, which in turn initiates a signaling cascade leading to various cellular responses. GPCRs are involved in numerous biological functions, including sensory perception, neurotransmission, hormone regulation, immune response, and many others. Due to their importance in cellular signaling, GPCRs have become a primary focus for pharmaceutical drug development, with approximately 700 approved drugs specifically designed to target GPCRs [11].

GPCRs can be classified into several categories based on different criteria. One commonly used classification system is based on their sequence homology and evolutionary relationships, resulting in the division of GPCRs into six classes or families, the A–F system [12,13] (Table 1). These classifications provide insights into the structural and functional similarities among GPCRs. The largest and most characterized GPCR family is the rhodopsin-like family (Class A), which includes numerous receptors involved in sensory perception, neurotransmission, and hormone signaling. Examples of Class A GPCRs include adrenergic receptors, dopamine receptors, serotonin receptors, and muscarinic receptors [14]. Another major GPCR family is the secretin-like family (Class B), which comprises receptors for peptide hormones, growth factors (B1) and adhesion receptors (B2). These receptors often have a long N-terminal extracellular domain of 120–160 residues that initiates the hormone peptide’s C-terminus recognition. This initial “first step” interaction orients the N-terminal region of the hormone peptides towards the J-domain of receptors, a structure formed by the upper parts of the transmembrane region and the three extracellular loops, thus resulting in a ‘second-step’ of ligand–receptor interaction that activates the receptor [15]. They play crucial roles in regulating various physiological processes, including metabolism, stress response, and immune function. The metabotropic glutamate receptor family (Class C) is another significant GPCR group. These receptors are involved in synaptic transmission and are characterized by their large extracellular N-terminal domain which comprises 500–600 residues and contains the orthosteric binding site of the receptor for the endogenous agonist [16]. Other GPCR families include the fungal mating pheromone receptors (Class D) and the frizzled/smoothened family (Class F). This last family is primarily involved in the regulation of developmental processes, particularly in embryonic growth and tissue patterning, playing a crucial role in shaping tissues and organs during embryogenesis and maintaining tissue integrity in adulthood [17]. Bitter taste receptors (Taste 2) have also been proposed to form an additional family (Class T) [18] (Table 1).

**Figure 1:**
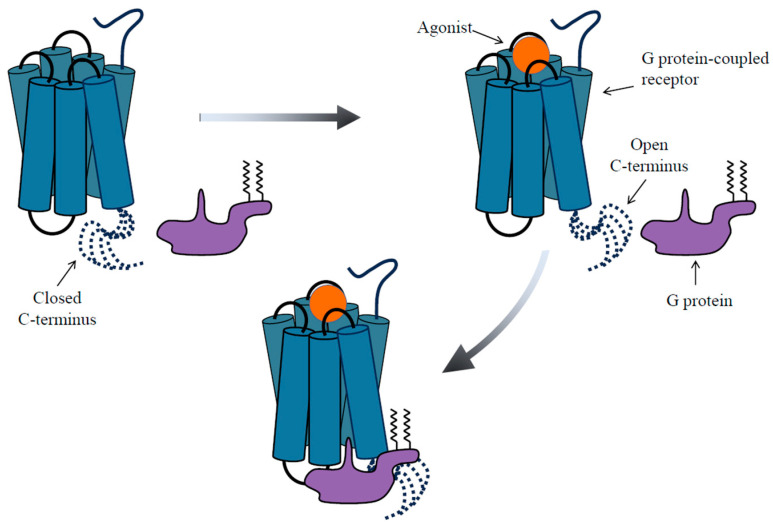
This model, greatly influenced by the research of Heng et al. [19], illustrates how the dynamic movement of a long and flexible C-terminus can modulate the accessibility of GPCR to the G protein. In the inactive state, the C-terminus assumes diverse conformations, engaging interactions with both the intracellular loops and the transmembrane core of the receptor, as indicated by the dashed lines in the upper-left corner. This conformation, referred to as the “closed C-terminus”, serves to restrict the receptor’s accessibility to the G protein. Upon binding of an agonist (or a positive allosteric modulator) to the receptor, a conformational shift occurs, weakening the interaction between the C-terminus and the receptor’s cytoplasmic surface. This shift leads to the displacement of the C-terminal region, allowing the G protein access to the receptor, referred to as the “open C-terminus”. In this state, the G protein can bind and initiate signaling. The dynamic interplay between the open and closed states of the C-terminus constitutes a previously unrecognized mechanism by which GPCRs finely regulate their coupling to G proteins.

Studies have demonstrated that the N- and C-terminus, along with the (i3) loop of GPCRs (Figure 1), typically have unstructured conformations.

As a result, these regions possess significant flexibility and are highly susceptible to interactions with various ligands, especially the N-terminus, and other protein partners. Our primary focus in this review will be the intracellular functions of the C-terminus and the i3 loop of GPCRs, while briefly touching upon the N-terminus as well.

## 3. The i3 Loop of GPCRs

The i3 loop is situated between the fifth and sixth transmembrane helices of GPCRs and is an important region that plays a crucial role in signal transduction and receptor regulation. The first function identified for the i3 loop was its interaction with G proteins, one of the major steps in GPCR intracellular signaling transduction. For example, in a study by Wess et al. [20], the muscarinic acetylcholine M3 receptor subtype was used as a model to identify a small amino acid sequence located at the N- and C-terminus of the i3 loop, that, along with the second intracellular loop (i2), was found to play a crucial role in the selective recognition of Gq/11 proteins. Significantly, the deletion of a substantial portion of the i3 loop (196 amino acids) in the M3 receptor did not affect its capacity to bind the G protein and to internalize. However, as we will elaborate on later, this deletion effectively hindered domain swapping between protomers of the M3 receptor dimer [21].

**Figure 2:**
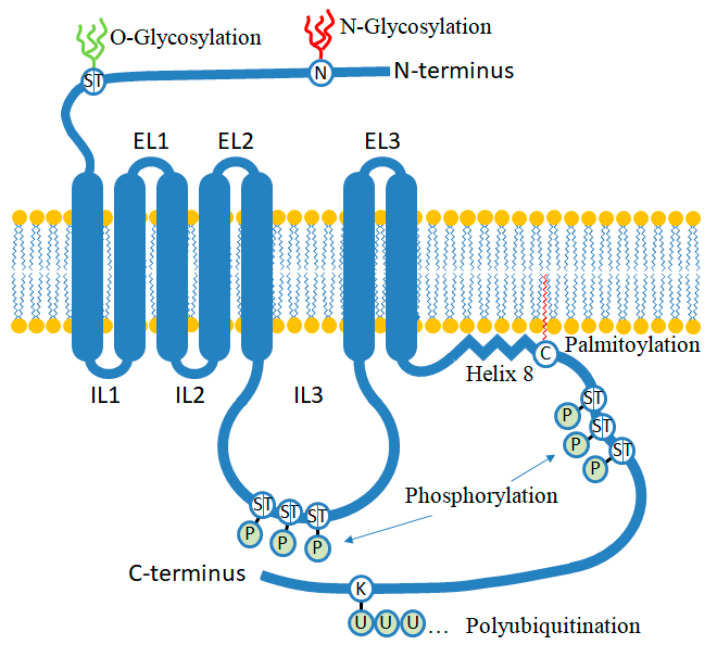
This scheme illustrates some of the post-translational modifications that occur in GPCRs. The N-terminus can undergo N- or O-glycosylation at asparagine (N) or serine (S)/threonine (T) sites, respectively. Phosphorylation (P) takes place either on intracellular loop 3 (IL3) or on the C-terminus at serine/threonine sites. When clusters of 3 serine or threonine residues within a span of 7 or 8 amino acids are phosphorylated, they are recognized by arrestin. Palmitoylation (red zigzag line) occurs at cysteine (C) residues in the C-terminus, resulting in the formation of an additional α-helix structure (Helix 8). Ubiquitination (U) occurs on specific lysine (K) residues, promoting protein transport to the proteasome and subsequent receptor degradation, or determining a specific trafficking route for the receptor within the cells. For more detailed information on post-translational modifications, you can refer to the review authored by Patwardhan et al. [26].

The i3 loop contains multiple phosphorylation sites targeted by protein kinases such as G protein-coupled receptor kinase (GRK), protein kinase A (PKA) and protein kinase C (PKC), which can promote recruitment of regulatory proteins such as arrestins [22]. Remarkably, the specific location of phosphorylation does not play a crucial role in the recruitment of arrestins, but rather, it is the increase in bulk negative charge that is the key factor in GPCR–arrestin interaction. Clusters of GRK phosphorylation sites are commonly found in most GPCRs. These clusters typically consist of three serine or threonine residues within a span of seven or eight amino acids, forming a phosphorylation code known as Px(x)PxxP, where P represents a phospho-serine or phospho-threonine, and x represents any amino acid residue except proline in the second occurrence of xx [23]. The presence of multiple phosphorylation events within this specific amino acid stretch is essential for the receptor to effectively bind arrestin (Figure 2). The recruitment of arrestin by the i3 loop enables the activation of alternative pathways distinct from G protein-mediated signaling and facilitates receptor desensitization and internalization [24]. It is interesting to note that different degrees in the i3 loop (and C-terminus) phosphorylation levels could have an impact in their downstream protein interactions to promote ligand, and cell type specific intracellular signaling [25]. For instance, differential expression, or activation of specific kinases such casein kinase 1 and 2 could significantly modify receptor functions; on the other hand, some ligands might instead modify receptor conformations to hide or expose phosphorylation sites that are necessary for arrestin and G protein interactions, leading to bias signaling.

The i3 loop displays significant variability in length and amino acid sequence across various GPCRs and even within different subtypes of the same receptor family. For instance, when considering the muscarinic receptor subtypes, the length of the i3 loop ranges from a minimum of 157 amino acids in the M1 receptor to 239 amino acids in the M3 receptor. Similarly, for dopamine receptors, the i3 loop length varies from a minimum of 50 amino acids in the D5 receptor to 160 amino acids in the D2 receptor (Table 2).

Interestingly, a distinct trend can be observed in many receptors, where there is an inverse relationship between the length of the i3 loop and the carboxyl terminus (Table 2). When the i3 loop is shorter, the carboxyl terminus tends to be longer, and vice versa. For example, the adrenergic α1A, α1B, and α1D receptor subtypes exhibit a short i3 loop and a long carboxyl terminus, whereas the adrenergic α2A, α2B, and α2C receptor subtypes demonstrate the opposite pattern, with a long i3 loop and a short carboxyl terminus (Table 2). This trend is not correlated with the signaling pattern of the receptors, as evidenced by the fact that the adrenergic α1 receptor subtypes, characterized by a short i3 loop, preferentially couple to Gq/11 proteins in a similar way to M1, M3, and M5 receptors, which possess a long i3 loop (Table 2). Conversely, there are many other receptors in which the i3 loop and carboxyl terminus are similar in length (see the five melanocorin receptors in Table 2). It is worth noting that the i3 loop and the C-terminus are not essential for the interaction between the G protein and GPCR. The critical element for G protein coupling resides in the cytoplasmic cavity formed between transmembrane helices, which undergoes conformational rearrangement during GPCR activation to accommodate the G protein [27].

The i3 loop of GPCRs exhibits a remarkable level of structural flexibility and lacks a well-defined secondary structure. Its interaction with other protein partners primarily relies on specific sequences of amino acids dispersed throughout this segment, rather than the three-dimensional conformation it adopts within the GPCR. This conclusion is supported by several lines of evidence demonstrating that targeted deletions within this loop result in functional impairment. Apart from its role in interacting with arrestins, the deletion of specific segments of the i3 loop can impact the interaction of GPCRs with various other proteins. Examples include regulators of G protein signaling (RGS) proteins [28], and endophilin proteins involved in cellular trafficking [29], as well as TULP3, RABL2, and the BBSome complex adapter protein, which are crucial for GPCR transport in cilia [30]. These are just a few examples of the protein interactions affected by alterations in the i3 loop; however, it is important to note that a significant portion of the i3 loop is not essential for these interactions, suggesting that its overall length likely serves other purposes, as will be discussed later.

## 4. The C-Terminus of GPCRs

The C-terminus of GPCRs is connected to the seventh transmembrane helix on the intracellular side of the plasma membrane. Indeed, it can bind to the cell membrane through a lipid modification that involves the attachment of a fatty acid, typically palmitic acid, to cysteine residues within its sequence. Palmitoylation serves as a lipid anchor, leading to the formation of an α-helix (usually called helix 8). Helix 8, in contrast to membrane-spanning seven helices of the GPCR core, is parallel to the membrane [31]. This helix structure contributes to the stability and proper functioning of the GPCR, while also influencing interactions with intracellular proteins and downstream signaling pathways [31].

Similarly to the i3 loop, the C-terminal region of GPCRs plays a critical role in various aspects of GPCR function and regulation. It harbors sites for post-translational modifications, such as phosphorylation, which are crucial for recruiting regulatory proteins involved in receptor trafficking, internalization, and degradation [32]. Additionally, the C-terminal region can contain binding sites for intracellular signaling proteins, adaptor proteins, and other regulatory molecules, enabling the receptor to engage with diverse intracellular signaling pathways, similar to the i3 loop [33,34]. The binding of PDZ domains, which are primarily suited for binding the distal regions of receptor C-terminal tails, represents a notable characteristic of the C-terminus [35]. They are structural modules involved in protein–protein interactions, specifically recognizing C-terminal peptide sequences and facilitating the assembly of intracellular complexes, by anchoring receptor proteins in the membrane to cytoskeletal components [36,37].

In the same way as the i3 loop, the C-terminal region of GPCRs exhibits large variations in length and amino acid sequences across different GPCRs and even within subtypes of the same receptor family. If we consider the dopamine receptors, belonging to the rhodopsin family of GPCRs, we observe a range in the number of amino acids of the C-terminus, varying from about fifteen amino acids for dopamine D2, D3, and D4 receptors, to over one hundred amino acids for dopamine D1 and D5 receptors (Table 2). The primary amino acid sequence leads to an inherently disordered and remarkably flexible conformation of this receptor segment [38]. As discussed earlier for the i3 loop, the C-terminal region of GPCRs primarily engages with other protein partners through specific amino acid sequences scattered across this region, rather than relying on its three-dimensional conformation within the GPCR [33].

## 5. The i3 Loop and the C-Terminus Play Significant Roles in Allosterically Regulating GPCR Activation

Multiple lines of evidence have demonstrated that the deletion of a substantial portion of either the i3 loop or the C-terminus seemingly does not alter the capacity of GPCRs to activate G proteins or bind arrestins [21,39]. This would suggest that these unstructured protein segments may not be indispensable for the normal functioning of the receptor. Despite the previously mentioned findings, two recent articles provide compelling evidence that these segments play crucial roles in regulating receptor function, as their folding significantly impacts the downstream signal transduction process following receptor activation [19,40]. In particular, Sandler et al. [40] demonstrated that the i3 loop of the β2 adrenergic receptor acts as an autoregulator of receptor activity by dynamically adopting different conformations that either block or expose the receptor’s G protein-binding site. Moreover, their study revealed that the i3 loop contributes to signaling specificity by inhibiting receptor coupling to G protein subtypes that have a weaker affinity for the receptor. Remarkably, this negative selection mechanism involving the i3 loop plays a pivotal role in fine-tuning G protein coupling selectivity. Specifically, the i3 loop buffers weakly coupled receptor–G protein interactions, which are poorly compatible with the receptor’s cytosolic G protein-binding interface, thereby reinforcing the coupling with cognate G proteins. This effect arises from a dynamic equilibrium between closed state of the l3 loop, which obstructs the G protein-binding site and renders the receptor inactive, and open state of the i3 loop, which allows for receptor–effector interactions promoting G protein-dependent signaling. Despite the significant diversity in the sequence of i3 loops, their experimental and bioinformatic analyses consistently indicate that a length threshold of approximately 45 amino acids serves as a critical determinant for gating G protein selectivity.

Conducting research on five Gs-coupled receptors with a long C-terminus, including adrenergic β2, adenosine A2A, dopamine D1 and D5, and serotonin 5-HT6 receptors, Heng et al. [19] revealed the C-terminus to exert direct inhibition over basal and agonist-stimulated G protein signaling. This inhibitory effect was attributed to the negatively charged nature of parts of the C-terminus, which acts as an auto inhibitory element by interacting with the positively charged cytoplasmic surface of i2 and i3 loops of the receptor, thereby restricting G protein access (Figure 1). Interestingly, the stability of this interaction was subject to modulation by agonists and allosteric modulators. Specifically, the binding of agonists weakens the interactions between the C-terminus and the cytoplasmic surface, ultimately enhancing the receptor’s accessibility to Gαs proteins. This highlights the crucial role of the C-terminus in the allosteric regulation of GPCR activation.

These two studies provide the first conclusive evidence regarding the regulatory roles of the i3 loop and the C-terminus in receptor function. The elongation of these segments, regardless of their amino acid sequence, creates steric hindrance that enables the receptor to selectively couple with G proteins to which they possess a high affinity. Nonetheless, if these were the primary function of these segments, evolution would likely have narrowed the differences in their lengths, resulting in a more uniform extension. Therefore, it is plausible that the length of these segments serves additional functions beyond the one described above.

The phenomenon described above, pertaining to the effect of the i3 loop and C-terminus restricting the accessibility of the receptor to G proteins, resembles the competitive effect observed in some receptor where the unstructured N-terminus plays a role as an agonist [41]. For instance, in the case of melanocortin MC4 receptor, a highly conserved six-amino-acid peptide at the N-terminus, confers constitutive activity to the receptor by binding deep inside the transmembrane regions 5 and 6 [42]. Note that this differs significantly from the constitutive activity imparted by the N-terminus to the protease-activated receptor (PAR) or to the adhesion GPCRs, where proteolysis of the N-terminal end releases the tethered agonist [43,44]. Notably, this N-terminus-mediated agonism competes with agouti-related peptide (AgRP), an endogenous MC4 receptor inverse agonist that reduces the constitutive activity of the receptor [45]. These findings suggest that evolutionary processes have orchestrated similar mechanisms to regulate the interaction of GPCRs with partner proteins on both the extracellular and intracellular sides. This remarkable convergence highlights the importance of these regulatory mechanisms in ensuring precise and sophisticated cellular signaling through GPCRs.

It is crucial to note that this regulatory mechanism does not apply to GPCRs with very short i3 loops and C-termini. In such cases, these GPCRs maintain a common theme, where all proteins that preferentially bind to activated GPCRs (such as G proteins, arrestins, and GRKs) utilize the cytoplasmic cavity formed between transmembrane helices, which undergoes conformational rearrangement during GPCR activation. The pivotal role of this cavity is substantiated by the structural analysis of GPCRs complexed with G proteins [27], arrestins [46], and the recent structure of GRK1 bound to rhodopsin [47].

## 6. Implications of the i3 Loop in Domain Swapping

Domain swapping is a process involving the dissociation of a domain from its parent protein and its subsequent association with another identical protein or a homologous protein in the same or different quaternary structure. Domain swapping has been identified in a diverse range of proteins, spanning enzymes, receptors, and other functional proteins. The phenomenon is thought to play a critical role in protein oligomerization, conformational changes, and functional regulation [48]. A region of protein, termed “hinge” loop, facilitates the swapping process.

**Figure 3:**
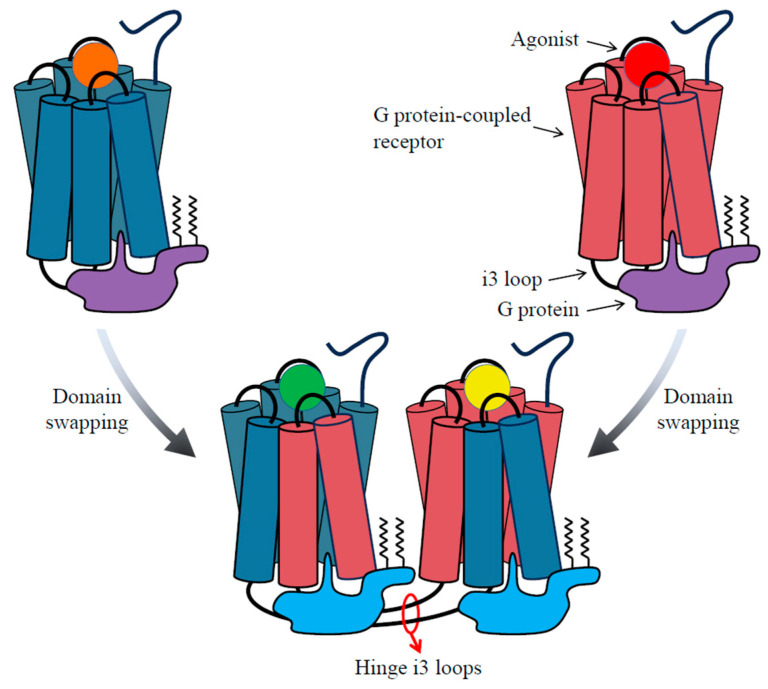
This illustration represents the hypothetical mechanism underlying domain swapping between two GPCRs. This proposed mechanism primarily pertains to receptors within the same subtypes, such as dopamine D2 and D3, as well as muscarinic M2 and M3 receptors. The process involves the exchange of transmembrane regions VI and VII between these two receptors, visually portrayed by the interchange of the cylindrical segments in the diagram. This intriguing phenomenon can result in either a modification of drug selectivity, evident through the distinct colors denoting ligands bound to the receptors, or an alteration in G protein coupling selectivity. This selectivity shift is visualized by the differing colors representing G proteins bound to the receptors, whether in their monomeric or heterodimeric forms. It is worth noting that this model, as well as class A GPCR dimerization per se, is highly controversial. In fact, all structures of the complexes of GPCRs with G proteins, arrestins, and of rhodopsin with GRK1, reveal a single GPCR bound to one molecule of a signaling protein. Class C GPCRs are true dimers, but they function without domain swapping. Nevertheless, as indicated in the text, the phenomenon of domain swapping has been leveraged in vivo to rescue the function of defective GPCRs.

While still a matter of controversy, domain swapping has indeed been observed in GPCRs. A prominent illustration of this phenomenon in GPCRs relates to the i3 loop, serving as a hinge connecting transmembrane regions V and VI, thus enabling the interchange of upstream and downstream segments between receptors (Figure 3).

The initial evidence of this phenomenon was presented by Maggio et al. [49], who engineered two chimeric receptors, α2/M3 and M3/α2, by exchanging the C-terminal portions of the receptors containing transmembrane domains VI and VII between the α2C-adrenergic and the M3 muscarinic receptor. When COS-7 cells were transfected with either of the two chimeric constructs individually, no detectable binding activity was observed. However, the co-transfection of α2/M3 and M3/α2 led to the emergence of specific binding sites for adrenergic and muscarinic ligands and a pronounced functional activity. Subsequently, it was demonstrated that this phenomenon is contingent on the length of the i3 loop, as shortening this segment prevented domain swapping [50].

In principle, the domain swapping between different GPCRs has the potential to generate novel receptors with distinct binding and coupling characteristics compared to the native receptors [51]. Furthermore, this phenomenon can impact receptor oligomerization, thereby influencing receptor trafficking, stability, and subcellular localization [52]. Domain swapping, wherein the i3 loop acts as a hinge, has been demonstrated between muscarinic M2 and M3 receptors [21,39], dopamine D2 and D3 receptors [53,54] and histamine H1 receptors [55]. The primary concern regarding these studies is that domain swapping was observed between mutant receptor monomers that exhibited either binding defects or non-functionality. This observation suggests that the domain exchange might have been compelled by the low affinity of the domains in the mutant monomers and may not have occurred with wild-type receptors. However, this issue remains unanswered as no definitive experiments have been conducted to either confirm or refute this phenomenon with wild-type receptors.

Notwithstanding this limitation, the phenomenon of domain swapping has been leveraged in vivo to rescue the function of defective GPCRs. As an illustration, using the mouse luteinizing hormone receptor (LHR) as a model GPCR, Rivero-Müller et al. [56] demonstrated that transgenic mice coexpressing binding-deficient and signaling-deficient forms of LHR could restore normal LH actions through intermolecular functional complementation of the mutant receptors in the absence of functional wild-type receptors. These findings offer compelling evidence for the in vivo occurrence of the domain swapping mechanism in GPCR signaling, albeit with engineered GPCRs.

## 7. Is the Length of the Unstructured C-Terminus and i3 Loop Crucial in Providing Flexibility and Expanding the Capture Radius for GPCR Interactions with Intracellular Protein Partners?

As mentioned earlier, the free COOH-terminal peptide of the C-terminus of several GPCRs have the ability to bind PDZ domains [57,58]. PDZ domains comprise around 90 amino acid residues that are characterized by a region of sequence homology, and they are present in single or multiple copies predominantly in cytoplasmic proteins. Traditionally, the specificity of PDZ interactions has been attributed to the last three residues of the protein C-terminus. Two consensus sequences have been proposed based on binding motifs: “S/T–X–Φ” and “Φ–X–Φ” (where X is any amino acid and Φ represents any hydrophobic residue), respectively, for class I and II PDZ domains, as grouped by Songyang et al. [59]. However, this classification system has recently faced challenges due to the discovery that certain PDZ-binding sequences do not conform to either class I or class II. Additionally, observations have shown that certain PDZ domains can bind promiscuously to both class I and class II ligands. Furthermore, it has become evident that residues located upstream of the last three amino acids also contribute significantly to the specificity of PDZ interactions [36,60].

**Figure 4:**
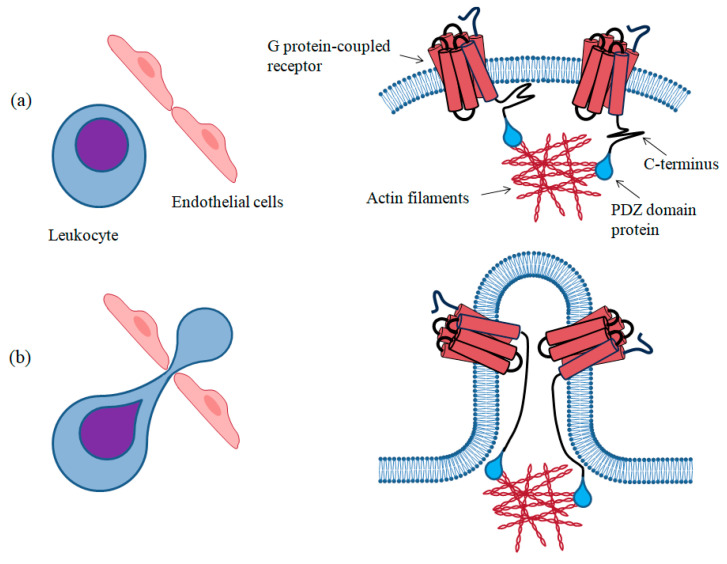
This picture illustrates how a long and flexible C-terminal can effectively anchor GPCRs to the underlying scaffolding proteins. In the upper-left of the illustration (**a**), a resting leukocyte is depicted as a round cell circulating in the bloodstream. In this state (upper-right), the two GPCRs can interact with the underlying actin filaments seamlessly due to their C-terminus and the PDZ domain protein bridging this interaction. Moving to the lower-left of the illustration (**b**), a leukocyte responds to a chemotactic stimulus and begins to cross the endothelial barrier of the blood stream. As the leukocyte crosses this barrier, its membrane must adapt to the narrow slit between endothelial cells, leading to its slow extravasation from the bloodstream. In the lower-right part of the illustration, the leukocyte’s membrane bends to form a loop that contains the two receptors. The flexibility and length of their C-terminus enable the receptors to stay firmly anchored to the actin filaments, maintaining the same spatial arrangement even after the leukocyte has crossed the endothelial barrier. It is worth noting that a tight fold or a shorter length of the C-terminus would have weakened the receptor’s interaction with the underlying scaffold, drastically altering the spatial compartmentalization of the GPCRs on the cell membrane. The illustration exaggerates the phenomenon but provides a representative understanding of the actual occurrence.

The unique positioning of this motif at the extreme end of the GPCR’s C-terminus, associated with the length of this unstructured segment, allows for an expanded radius of interaction. This, in turn, facilitates the receptor’s binding to PDZ domains and, for instance, its interaction with structural proteins beneath the membrane surface. An illustrative example of this phenomenon involves the binding rate of a specific protein segment within an unfolded protein, which can be notably higher than the rate of binding of the same segment within a fully folded protein. This difference arises due to the smaller fluctuation exhibited by the folded protein [61]. Additionally, the flexibility of the C-terminus enables increased adaptability in interactions, ensuring the GPCR remains firmly anchored to the underlying structure even during the cell membrane’s natural movements. For example, this phenomenon is particularly pronounced during cell migration and extravasation, where the plasma membrane undergoes a significant stretch to enable the cell’s passage across the endothelium (Figure 4).

Furthermore, the flexibility of the C-terminus may play a significant role in enabling GPCR to internalize and to be sorted to different intracellular compartments [10], as GPCRs will follow the curvature of the membrane remaining attached to the surrounding scaffolding proteins. A more rigid C-terminus, resulting from tight packing, or a shorter length of this segment, could weaken the connection between the GPCR and the scaffold surrounding the membrane, leading to the release of the receptor and potentially preventing proper intracellular sorting.

Apart from protein containing PDZ domains, this rationale can be significantly extended to encompass other proteins that interact with the C-terminus of the GPCRs. Additionally, it is plausible that in proteins interacting with the GPCRs at multiple sites other than the C-terminus, the initial interaction could occur with the C-terminus, that subsequently guides the protein to its final position. For example, arrestins typically associate with GPCRs at the phosphorylated C-terminus (or i3 loop). Furthermore, arrestins bind to the cavity that opens upon receptor activation on the intracellular side between transmembrane helices V and VI, which move outward. This final interaction holds pivotal significance in terminating G protein-coupled receptor signals, as arrestins and G proteins engage in a competitive binding process for the same cavity [62].

The recruitment of arrestin by a GPCR has been proposed to begin with the interaction between inactive arrestin and the phosphorylated receptor C-tail. This interaction converts the inactive arrestin into a pre-activated state, enabling it to bind to the GPCR with high affinity. Electron microscopic studies of the β2 adrenergic receptor and vasopressin V2 receptor C-tail chimeric receptor complexed with β-arrestin-1 revealed that β-arrestin-1 can bind to either the central cytoplasmic cavity of the receptor TM bundle in a fully engaged state or to the receptor’s C-terminal tail in a partially tail-engaged state [63,64]. Based on these findings, Shukla et al. [58] proposed a model in which arrestins likely employ a biphasic mechanism to interact with the receptor. In the first phase, there is an interaction between the phosphorylated C-terminal tail of the receptor and the N-terminal domain of arrestin. Due to its flexibility and length the C-terminal receptor tail is expected to act like a fishing line, exploring a wide interaction space at a high rate. This initial interaction will then guide arrestin to the second point of interaction, which seems weaker and primarily involves the insertion of the finger loop of arrestin within the receptor core. This final arrangement would likely prevent GPCR engagement with G protein heterotrimers, leading to the blockade of classical GPCR signaling and triggering desensitization. As emphasized earlier, it is crucial to bear in mind that numerous GPCRs exhibit strong binding with wild-type arrestins even in the absence of receptor phosphorylation.

This fishing line effect is exemplified by the so-called “fly-casting mechanism” that refers to the idea that intrinsically disordered regions of proteins can increase the rate of binding to their target molecules by extending and sampling a larger conformational space, effectively “casting” for interactions like a fly fisherman casting a line [61,65]. The fly-casting mechanism can enhance the kinetics of binding between proteins. Since intrinsically disordered regions can rapidly sample different conformations, they can bind to their target molecules more quickly than if they had a fixed, structured shape. This is especially important in cases where the binding partner may be initially distant or where the binding site is partially occluded.

Similarly, the length of the unstructured i3 loop in GPCRs might also play a crucial role in providing flexibility and expanding the capture radius for interactions with intracellular protein partners. As observed with the C-terminus, the i3 loop may possess unique characteristics that allow it to interact with specific proteins, including protein containing PDZ domains such as spinophilin [66]. These interactions could be facilitated by the length of this unstructured segment, which will allow for an expanded radius of interaction. Like the C-terminus, the flexible nature of the i3 loop could enable GPCRs to adapt and firmly bind to underlying structural proteins, even during dynamic movements of the cell membrane.

A comparable fishing line effect can be observed in the unstructured extracellular N-terminus of chemokine receptors exemplified by CXCR1 [67]. The extracellular N-terminal domain of CXCR1 acts as the primary binding site for the endogenous ligand, interleukin 8 (CXCL8). The notable extension and flexibility of this N-terminal segment create a fishing line effect, enhancing the probability of capturing and tethering CXCL8. Upon binding to CXCL8, the N-terminal domain guides the ligand to the second binding site on the extracellular loops, potentially causing a conformational change in CXCR1 and leading to subsequent G protein activation.

## 8. The Role of the C-Terminus in the Retrotranslocation of GPCRs

Retrotranslocation is a crucial mechanism that prevents newly synthesized misfolded proteins from exiting the endoplasmic reticulum (ER) and reaching their final destinations. This process begins with the identification of misfolded proteins within the ER, followed by their retrotransport into the cytosol for subsequent degradation through the ubiquitin–proteasome system. This regulatory pathway is referred to as ER-associated protein degradation (ERAD) [68]. While its initial conception pertained to the mere removal of misfolded proteins to safeguard cellular survival, it is now recognized that this mechanism actively influences protein abundance by modulating their numbers. The initial observation of such a modulatory effect pertains to the inositol 1,4,5-trisphosphate (IP3) receptors, a tetrameric channel that regulates the release of Ca^2+^ ions stored within the ER lumen in response to the second messenger IP3 [69]. The structural alterations responsible for initiating channel opening cause IP3 receptors to adopt a conformation resembling misfolded proteins. This, in turn, prompts their interaction with the ER lipid raft protein (erlin)-1 and erlin-2 complex [70], followed by their ubiquitination and subsequent removal from the ER membrane. Ultimately, these receptors undergo degradation through the proteasome machinery. This degradation process of activated IP3 receptors could potentially shield cells from the harmful consequences stemming from the excessive activation of calcium signaling pathways [69]. Another receptor whose levels are influenced by this mechanism is the N-methyl-D-aspartate (NMDA)-type glutamate receptor, a ligand gated ion channel receptor that plays a pivotal role in synaptic plasticity [71]. In this instance, following retrotranslocation, the glycosylated and elongated N-terminal segment of the NMDA receptor engages with Fbx2, a neuron-specific F-box protein adept at identifying N-linked high-mannose oligosaccharides on substrate proteins [72]. This engagement serves to catalyze ubiquitination and subsequent degradation of the receptor.

When examining GPCRs, this mechanism was first demonstrated with the δ opioid receptor (DOR). In this context, a significant portion of newly synthesized receptors initially remains confined within the ER, where they undergo retrotranslocation and subsequently become marked for degradation [73]. This mechanism showed that the ER quality control can eliminate a high proportion of wild-type receptor proteins. Furthermore, retrotranslocation plays a role in the downregulation induced by agonists of μ opioid (MOR) and DOR receptors [74]. Interestingly, pre-treating cells with lysosomal protease inhibitors showed minimal impact on the downregulation of μ and δ opioid receptors, suggesting that the internalized receptors are not subject to degradation within lysosomes. In contrast, pretreatment with proteasome inhibitors mitigated the agonist-induced downregulation of μ and δ receptors, indicating that these receptors undergo retrotranslocation followed by proteasome-mediated degradation. Other GPCRs that have been explored in this context include the thyrotropin-releasing hormone receptor (TRHR) [75] and the yeast pheromone receptors [76]. Notably, both of these receptors undergo modification through the addition of ubiquitin residues to their C-terminal regions, triggering their retrotranslocation and subsequent degradation. These findings underscore an additional conceivable function of the unstructured C-terminal segment of GPCRs in instigating an atypical form of receptor downregulation, orchestrated through ubiquitination, retrotranslocation into the cytosol, and subsequent degradation, as opposed to the conventional route involving lysosomal pathways [77].

## 9. Conclusions

The study of unstructured regions in functional proteins has gained significant attention due to advancements in informatics tools and biophysical methods. In the context of GPCRs, the i3 loop and C-terminus have been identified as regions with intrinsic disorder, providing them with significant flexibility and susceptibility to interactions with various ligands and protein partners. This review highlights the emerging understanding of the functional significance of these unstructured regions in GPCRs.

Recent studies have demonstrated that the i3 loop and C-terminus are not merely accessory regions but play significant roles in allosterically regulating GPCR activation. The i3 loop, acting as an autoregulator, dynamically adopts different conformations that either block or expose the receptor’s G protein-binding site, impacting G protein coupling specificity. The C-terminus directly inhibits basal and agonist-stimulated G protein signaling through interactions with the cytoplasmic surface, with agonists weakening this interaction and enhancing receptor–G protein accessibility. These findings suggest that the i3 loop and C-terminus provide critical regulatory mechanisms to fine-tune GPCR signaling.

The unstructured nature and length of the i3 loop and C-terminus offer unique advantages in the interactions between GPCRs and intracellular protein partners. The fishing line effect of these segments allows for an expanded radius of interaction, enabling GPCRs to tether to scaffolding proteins and remain anchored during cell membrane movements. Furthermore, the unstructured i3 loop might facilitate domain swapping between GPCRs, generating novel receptor dimers with distinct binding and coupling characteristics. Ultimately, the C-terminus is gaining recognition as a vital regulatory component in the retrotranslocation and degradation of GPCRs through its involvement in the ubiquitination process.

In conclusion, the i3 loop and C-terminus of GPCRs are now recognized as crucial players in receptor function and regulation. Their unstructured nature and flexibility offer unique advantages in receptor activation, signaling, interaction with intracellular partners, trafficking and degradation. Understanding the functional roles of these unstructured regions sheds light on the complexity of GPCR signaling and opens new avenues for research in receptor pharmacology and drug development.

## Figures and Tables

**Table 1:** Classification of GPCRs according to the GPCR database information system (https://gpcrdb.org/ accessed on 15 September 2023).

Family	Subtypes
Class A Rhodopsin	Aminergic receptors—Peptide receptors—Protein receptors—Lipid receptors—Melatonin receptors—Nucleotide receptors—Steroid receptors—Alicarboxylic acid receptors—Sensory receptors—Olfactory receptors—Orphan receptors—Other
Class B1 Secretin	Peptide receptors
Class B2 Adhesion	Adhesion receptors
Class C Glutamate	Ion receptors—Amino acid receptors—Sensory receptors—Orphan receptors
Class D1 Ste2-like	Ste2—like fungal pheromone receptors
Class F Frizzled	Protein receptors
Class T Taste2	Sensory receptors
Other GPCR	Orphan receptors

**Table 2:** Amino acid extent of the i3 loop and C-terminus within select representative GPCRs.

GPCR	Total AA	i3 Loop AA	C-Ter. AA	GPCR	Total AA	i3 Loop AA	C-Ter. AA
D_1_	446	54	109	α_1A_	466	67	137
D_2_	443	160	12	α_1B_	520	71	169
D_3_	400	120	14	α_1D_	572	73	167
D_4_	419	133	16	α_2A_	465	156	21
D_5_	477	50	117	α_2B_	450	175	24
				α_2C_	462	148	21
M_1_	460	157	40	β_1_	477	71	100
M_2_	466	178	24	β_2_	413	54	84
M_3_	590	239	44	β_3_	408	67	61
M_4_	479	184	23				
M_5_	532	229	34	MC_1_	317	29	17
				MC_2_	297	18	19
5HT_1A_	422	128	19	MC_3_	323	35	22
5HT_1B_	390	87	19	MC_4_	332	33	28
5HT_1D_	377	85	17	MC_5_	325	28	28
5HT_1E_	365	89	18				
5HT_1F_	366	89	16	A_1_	326	34	34
5HT_2A_	471	70	87	A_2A_	412	36	122
5HT_2B_	481	85	99	A_2B_	332	32	41
5HT_2C_	458	76	87	A_3_	318	33	34
5HT_4_	388	47	73				
5HT_5A_	357	63	16	CXCR_1_	350	22	42
5HT_6_	440	57	120	CXCR_2_	360	20	45
5HT_7_	479	68	91	CXCR_3_	368	22	47
				CXCR_4_	352	25	50
CB_1_	472	45	73	CXCR_5_	372	19	47
CB_2_	360	31	59	CXCR_6_	342	16	49

Total AA, i3 loop AA and C-ter. AA represent the total number of amino acids in the protein, in the i3 loop and in the C-terminus, respectively. GPCRs are identified by the following abbreviations: D = dopamine; M = muscarinic; 5HT = 5-hydroxytryptamine; CB = cannabinoid; α and β = adrenergic; MC = melanocortin; A = adenosine; CXCR = chemokine; Data were taken from UniProt (https://www.uniprot.org/ accessed on 14 August 2023).
